# A joint climate and nature cure: A transformative change perspective

**DOI:** 10.1007/s13280-021-01679-8

**Published:** 2022-01-25

**Authors:** Graciela M. Rusch, Jesamine Bartlett, Magni Olsen Kyrkjeeide, Ulrika Lein, Jenni Nordén, Hanno Sandvik, Håkon Stokland

**Affiliations:** 1 Norwegian Institute for Nature Research, Torgarden, P.O. 5685, 7485 Trondheim, Norway; 2 Norwegian Institute for Nature Research, Sognsveien 68, 0855 Oslo, Norway

**Keywords:** Biodiversity conservation, Climate mitigation, Cross-sectoral integration, Informed decisions, Natural climate solutions

## Abstract

Climate change has considerably dominated science-policy dialogue, public debate, and subsequently environmental policies since the three “Rio Conventions” were born. This has led to practically independent courses of action of climate change mitigation and biodiversity conservation actions, neglecting potential conflicts among outcomes and with missed opportunities for synergistic measures. Transformative governance principles have been proposed to overcome these limitations. Using a transformative governance lens, we use the case of the Norwegian "Climate Cure 2030" for the Land Use, Land-Use Change and Forestry (LULUCF) sector to, first, illustrate the mechanisms that have led to the choice of climate mitigation measures; second, to analyze the potential consequences of these measures on biodiversity and greenhouse gas (GHG) emissions; and, third, to evaluate alternative measures with potential positive outcomes for biodiversity and GHG emissions/removals. We point to some mechanisms that could support the implementation of these positive actions.

**Supplementary Information:**

The online version contains supplementary material available at 10.1007/s13280-021-01679-8.

## Introduction

Climate change is widely recognized as a leading environmental concern, a key driver of biodiversity loss, and it poses major societal challenges, including threats to the global economy (World Economic Forum 2020). These challenges have been recognized by the global community through the Paris Agreement (UNFCCC 2015) in an effort to address the underlying causes of climate change, namely greenhouse gas (GHG) emissions generated by the burning of fossil fuels, and land-use change. At the same time, the first Global Assessment by the Intergovernmental Science-Policy Platform for Biodiversity and Ecosystem Services (IPBES) on the status of nature and its contributions to people (IPBES 2019), confirmed that environmental challenges go far beyond those of climate change. The report stated that “the biosphere is being altered to an unparalleled degree across all spatial scales”, concluding that, worldwide, the greatest threat to terrestrial biodiversity is land-use change, i.e. the destruction and fragmentation of habitats and the biological homogenization of landscapes and ecosystems (e.g.McKinney and Lockwood 1999; Jongman 2002). Despite good intentions, little progress has been achieved in the past decade, and many of the 20 Aichi Biodiversity Targets under the Strategic Plan for Biodiversity 2011–2020 (Convention on Biological Diversity 2010) have been missed. Targets that include actions involving cross-sectoral coordination show particularly poor progress (IPBES 2019), viz. ‘harmful subsidies eliminated’ (3.1), ‘positive incentives developed and implemented’ (3.2), ‘sustainable production and consumption’ (4.1), ‘habitat loss at least halved’ (5.1), ‘degradation and fragmentation reduced’ (5.2), ‘excess of nutrients not detrimental’ (8.2), ‘invasive species controlled or eradicated’ (9.3), ‘invasive introduction pathways managed’ (9.4), ‘extinctions prevented’ (12.1), ‘conservation status of threatened species improved’ (12.2), and ‘ecosystems providing services restored and safeguarded’ (14.1).

It is not only species that are threatened, but also the ecological functions that underpin multiple ecosystem services. Since 1970, 14 of the 18 ecosystem services assessed in the Global Assessment have declined, including air quality regulation, water purification, climate stabilization, control of natural hazards, pollination, and pest outbreak control (IPBES 2019).

### Biodiversity and climate change in the global environmental political agenda

The challenges to protect nature and manage ecosystems sustainably have been a political priority, at least since the United Nations Conference on Environment and Development, in Rio de Janeiro 1992. Here, the three “Rio Conventions” were born, i.e. the UN Convention on Biological Diversity (CBD), the UN Framework Convention on Climate Change (UNFCCC) and the UN Convention to Combat Desertification (UNCCD). It has been the aim that the three conventions are intrinsically linked to develop synergies on issues of mutual concern (UNFCCC 2020). However, since its inception in 1994, the UNFCCC and its associated bodies and agreements have considerably dominated the science–policy dialogue and the public debate about the state and severity of, and the solutions to, global environmental challenges, to the extent that environmental challenges are often equated to climate change problems. For example, the Norwegian Environment Agency states that: “Our primary tasks are to reduce greenhouse gas emissions, manage Norwegian nature and prevent pollution”, indicating an urgency to stabilize the climate, but not in stopping biodiversity loss and the degradation of ecosystems and the services they generate.

The great impact of the UNFCCC, both on the level of concern and the actions needed to meet environmental challenges, has been attributed to the Convention following the line of the Montreal Protocol (on Substances that Deplete the Ozone Layer, 1987), which “bound the member states to act in the interests of human safety even in the face of scientific uncertainty” (UNFCCC 2020). An even greater influence has been the establishment of the Intergovernmental Panel on Climate Change (IPCC) in 1988, “to provide policymakers with regular scientific assessments on the current state of knowledge about climate change” (IPCC 2020). Since its foundation, the IPCC has produced six assessment reports at regular intervals, in addition to several thematic reports. The Nobel Peace Prize 2007 awarded to the IPCC evidences the enormous importance of climate change for society.

Two crucial and distinctive elements in the UNFCCC that have further contributed to the relatively rapid take-up and implementation of climate actions, are that the increase of atmospheric GHG can readily be attributed to human activities, and that it is possible to quantify the outcomes of climate mitigation measures and policies with a single index, the carbon dioxide equivalent (CO_2_-e), providing a common and transparent metric to monitor anthropogenic GHG emissions and removals by the biosphere.

Twenty-four years after the establishment of IPCC, IPBES was founded in 2012 to form a scientific body, equivalent in its function to the IPCC, with the aim to “strengthen the science-policy interface for biodiversity and ecosystem services for the conservation and sustainable use of biodiversity, long-term human well-being and sustainable development” (IPBES 2020).

### Addressing biodiversity and climate challenges simultaneously

Despite the alarming trends presented in the Global Assessment (IPBES 2019), the report concludes that “nature can be conserved, restored and used sustainably while simultaneously meeting other global societal goals through urgent and concerted efforts fostering transformative changes” across economic, social, political and technological factors, and points out that these changes need to address individual and societal values and behaviors, technological innovations and governance. Visseren-Hamakers et al. (2021) highlight that the discussion about how to govern such changes is still in its infancy and argue that transformative governance is needed to enable transformative change. They describe four transformative governance approaches that need to be implemented in conjunction to address sustainability issues: “*integrative*, to ensure local solutions also have sustainable impacts elsewhere (across scales, places, issues and sectors); *inclusive*, to empower those whose interests are currently not being met and represent values embodying transformative change for sustainability; *adaptive*, enabling learning, experimentation, and reflexivity, to cope with the complexity of transformative change; and *pluralist*, recognizing different knowledge systems”. These improved forms of governance would imply better coordination and implementation of actions to address intrinsically related societal challenges such as climate mitigation, and the conservation and sustainable use of biodiversity.

In this perspectives paper, we use Norway’s plan for carbon mitigation measures, Climate Cure 2030 (Norwegian Environment Agency 2020), which we consider as a pre-IPBES, ‘business-as-usual’ approach to solving climate change mitigation challenges, to illustrate current structural and procedural limitations to the transformative governance approaches proposed in Visseren-Hamarkers et al. (2021). This case serves as an example of how climate mitigation actions are a hindrance to the protection of biodiversity when one does not follow transformative governance approaches.

Following Visseren-Hamakers et al. (2021), we evaluate the proposed actions in terms of how:

(i) comprehensive knowledge from multiple sources is considered for decisions about climate mitigation and nature conservation (*pluralist*, through a revision of the knowledge base and its uptake in decision-making).
(ii) plans and measures foster cross-sectoral synergies and resolve conflicts between sectors that are detrimental for biodiversity and ecosystem services (*integrative* by showing how climate mitigation solutions consider possible synergies with biodiversity conservation and the generation of multiple ecosystem services);
(iii) value plurality and the due value of nature and its services are recognized when resolving trade-offs (*inclusive,* by illustrating how biodiversity conservation and ecosystem service priorities are incorporated in the climate action).

We do not evaluate the proposed measures in Climate Cure 2030 in terms of the fourth approach, i.e. how *adaptive* the governance approach is, and whether decisions are made transparently and regularly reviewed based on the best available knowledge and experience, but we bring in the ‘adaptive governance’ criteria, at a second stage, when we formulate options for the future (i.e. tools for monitoring and reporting on the effects of actions).

At a second stage, we suggest how limitations could be turned into positive actions if transformative governance approaches would be followed.

Our analysis first presents an overview of the knowledge base, and how it informs the selection of climate mitigation measures under the ‘Land Use, Land-Use Change and Forestry’ (LULUCF) sector. Second, we address how climate and biodiversity conservation and enhancement objectives are prioritized and harmonized within and across sectoral policies. Finally, we show the potential for new options that would embrace transformative governance approaches. We focus on LULUCF because of the potentially high level of conflict of climate mitigation actions with nature conservation objectives (IPBES 2019). These trade-offs, if not adequately addressed, will impair the joint achievement of multiple goals set under various multilateral agreements such as those under the CBD, the UNFCCC, the Gothenburg Protocol, and not least, the Sustainable Development Goals (SDGs), including SDGs 13 (Climate action), 14 (Life below water) and 15 (Life on Land).

### The Norwegian climate cure 2030

After the Kyoto protocol entered into force in 2005, the Norwegian Government’s climate-related policy development gained momentum (Norwegian Ministry of the Environment 2007). Since then, and following commitments with the Paris Agreement under the UNFCCC, the Norwegian Government has proposed a plan for the LULUCF consisting in three key measures (Box 1), called ‘*Climate Cure 2030*’ (Norwegian Environment Agency 2020). The goal of *Climate Cure 2030* is to increase the removals of atmospheric carbon by managed ecosystems (land-uses and forestry).

Box 1 – Not seeing the forest for the trees: The Norwegian Climate Cure 2030 *Climate Cure 2030* proposes three key measures for the LULUCF sector.

**Fig. 1:**
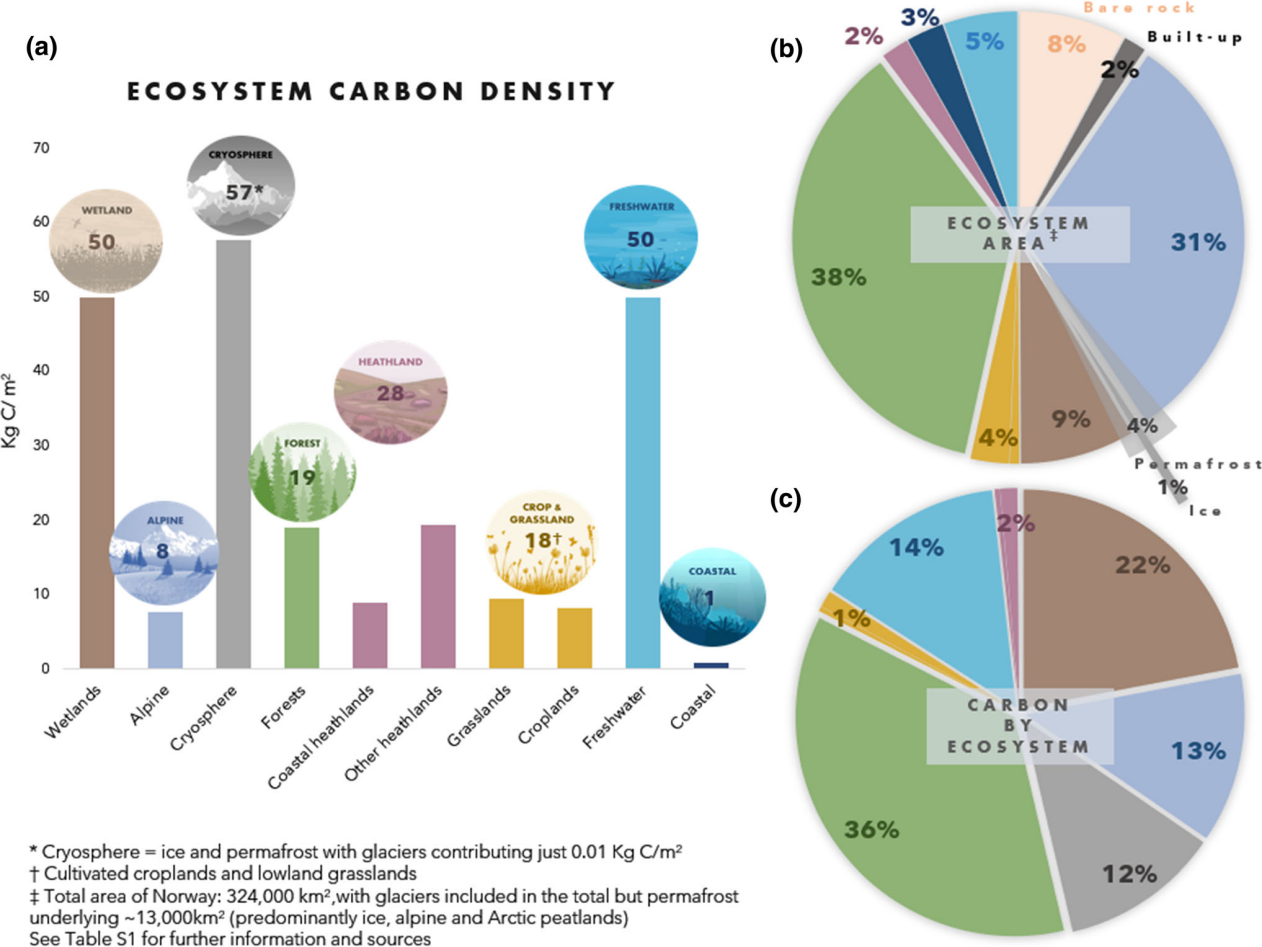
**a** Norwegian ecosystem carbon storage density (kg C m^−2^), shown with its constituent elements: **b** the area proportion (%) of ecosystem/land cover types; and **c** carbon storage estimates for each ecosystem, excluding ‘built-up’ and ‘bare rock’ area types, which do not have such data available. For communication purposes, lowland grasslands and cultivated land (crops) are color grouped, as are both ‘coastal’ and ‘other’ heathland types. All data and sources are detailed in Table S1

- **1. Increased density of trees in tree plantations**. Dense plantations may present high risks for Norwegian forestry in the face of climate change^1^ and provide little habitat for forest biodiversity. Dense plantations are also characterized by low cover of understorey vegetation, which contributes significantly to net carbon uptake^2^.
- **2. Forest fertilization applied 8–10 years before final felling**. Nitrogen fertilization encompasses risk for environmental pollution/eutrophication, reductions in plant and fungal diversity, changes in bacterial diversity, reduction in fungal biomass, and related changes in GHG emissions and carbon stocks^3,4,5^. Nitrogen fertilization can also increase the sensitivity of trees to drought and pest attacks by changing the level of chemical defence of conifer needles^6^.
- **3. Tree planting on non-forested land (afforestation)**. Can potentially have important negative effects on biodiversity in the case of open semi-natural habitats, which are critical areas for the conservation of light-demanding plant species and the organisms that use them. Grasslands, tree-less heathlands and wetlands have a very high potential to store soil carbon (Fig. 1), which has been largely underestimated. Also, tree plantation in these areas may not render the expected carbon removal level and could increase carbon loss and CO_2_ emissions^7^.

**1.** Swedish forest agency 2020. Climate adaptation of forest and forestry – goals and proposed actions (in Swedish). **2.** Wardle et al. 2012. Linking vegetation change, carbon sequestration and biodiversity: insights from island ecosystems in a long-term natural experiment. *J. Ecol.* 100:16–30. **3** Zhang, T.A., et al. 2018. Global negative effects of nitrogen deposition on soil microbes. *ISME* 12:1817–1825. **4.** Zhou, Z., et al. 2017. Patterns and mechanisms of responses by soil microbial communities to nitrogen addition. *Soil Biol. Biochem.* 115: 433–441. **5.** Midolo, G., et al. 2019. Impacts of nitrogen addition on plant species richness and abundance: A global meta-analysis. *Glob. Ecol. Biogeog.* 28:398–413. **6.** Nybakken, L., et al. 2018. Fertilization Changes Chemical Defense in Needles of Mature Norway Spruce (*Picea abies*). *Front. Plant Sci*. 9: 770. **7.** Brown, I. 2020. Challenges in delivering climate change policy through land use targets for afforestation and peatland restoration. *Env. Sci. Policy* 107: 36–45.

## The knowledge base for pluralist decision-making—the case of standing stock vs. ecosystem-wide management of carbon stocks

Pluralist governance requires a transformation in how societies value nature and its contributions. This builds on the increasing awareness of the importance of how evidence is collected and understood in informing and shaping decision-making (Jacobs et al. 2018).

Carbon accounting, i.e. how much and where carbon emissions and removals occur, is central to identifying measures to mitigate climate change. Due to the difficulty in providing widely applicable and scientifically robust methods to assess land-based GHG fluxes, the IPCC Guidelines have adopted a pragmatic approach, and GHG uptake in and emissions from “unmanaged land” are not reported in GHG inventories because they are assumed to be non-anthropogenic. Further, national GHG inventories rely on chosen methods of estimation and national accounting rules, which differ in approach and complexity among countries (Grassi et al. 2018).

Globally, all marine and terrestrial ecosystems are sinks for anthropogenic carbon emissions, with a gross sequestration of 5.6 Gt C year^−1^ (1 Gt = 1 gigaton = 10^12^ kg), the equivalent of some 60% of global anthropogenic CO_2_ emissions (IPBES 2019). Terrestrial plants and soils currently absorb approximately 33% of anthropogenic CO_2_ emissions, yet this is partially offset by emissions related to the land-use change (ca 10%) (Le Quéré et al. 2014). In terrestrial systems, up to 80% of the carbon (2500 Gt C) is found in soils, whereas the amount of carbon in living plants and animals is comparatively small (560 Gt C; Ontl and Schulte 2012; see Villa and Bernal 2018), highlighting the importance of functioning ecosystems for removing carbon from, and preventing release to, the atmosphere.

Norwegian ecosystems contain approximately 0.18% of all global carbon stocks, with a land mass that is 0.07% of that of the planet (Bartlett et al. 2020). This high carbon-to-area ratio is likely due to the large proportion of the country that is carbon-rich peatlands (alpine and lowland) and boreal forest. The largest stores of carbon are in forest habitats (32%) which also cover 38% of the total land area. Wetlands and permafrost-covered ecosystems, 9% and 3% of the total land mass, respectively (Bryn et al. 2018), store over 2.2 Gt C, 34% of the nation’s carbon (Fig. 1; Table 1). These two ecosystems are the most carbon dense terrestrial ecosystems, with an estimated 50 and 58 kg C m^−2^ for wetlands and permafrost, respectively. Similar carbon stores can be found in freshwater lake sediments, also with 50 kg C m^−2^, amounting to 14% of all carbon storage. Regarding the removal rate of atmospheric CO_2_, forests and low-mid alpine zones are estimated to take up the most carbon on an annual basis (5.5 and 5.3 Mt C year^−1^, respectively; 1 Mt = 1 megaton = 10^9^ kg), with soils in alpine heathlands contributing the most to alpine carbon stores.

**Table 1:** Carbon storage in Norwegian ecosystems

Ecosystem	C storage		Confidence		Accounting	Effect of land-use change
	In Mt C	In %	Evidence	Agreement		
Forests	2300	36	Limited	Medium	Estimated	High
Living trees	500	8	Robust	High	Measured	High
Understorey	23	< 1	Limited	High	Not included	High
Soil	1800	28	Limited	Low	Estimated	High
Alpine	800	12	Limited	Medium	If converted	Intermediate
Wetlands	1400	22	Limited	Medium	If converted	High
Freshwater	900	14	Limited	Medium	Not included	Low
Cryosphere	750	12	Limited	Low	If converted	Low
Open lowlands	200	3	Limited	Medium	Estimated	Intermediate
Cropland	78	1	Robust	High	Estimated	High
Grassland	22	< 1	Limited	High	Estimated	High
Heathland	100	2	Limited	Low	If converted	Intermediate
Coastal	8	< 1	Medium	Medium	Not included	Intermediate
Sum	6400	100	Limited	Medium	Estimated	High

*Confidence* in the carbon storage estimates is divided into type, amount and quality of *evidence*, and degrees of *agreement*. *Accounting* indicates whether and how this (sub)ecosystem is covered by the national LULUCF accounting system (either measured directly; estimated based on simplifying assumptions; calculated only for areas that are converted to or from an ecosystem type that is measured or estimated; or not included at all). *Effect on land-use change* refers to the effect that land-use changes may have on the ecosystem’s capacity to take up and store carbon. The underlying data and sources are provided in the Supplementary Information

## Not seeing the woods for the tree—a skewed knowledge base for carbon accounts in LULUCF

Despite the apparent simplicity of applying CO_2_-e as a universal metric of emissions and removals of atmospheric GHG, an accurate assessment of these values and how they are modified by ecosystem management is difficult, because it entails a systemic approach and the understanding of complex ecological processes. The fact that there are significant mismatches between measured atmospheric GHG concentrations and reported emissions and removals (Peters et al. 2017) indicates that there are considerable uncertainties and potential biases in the carbon removal assessments and reporting (Table 1).

Current carbon-removal and -emission accounts in the Norwegian LULUCF sector are based on data from an extended National Forest Inventory (NFI) program, which are skewed in terms of how different ecosystems’ carbon stocks are valued, which elements of the terrestrial carbon budget are considered, and how they are estimated. The IPCC LULUCF classes are forest, cropland, grassland, wetlands, settlements and other land. ‘Other land’ (4F) is defined in the NFI “as waste land, such as bare rocks, ice, and shallow soils that may have particularly unfavorable climatic conditions”. In accordance with the IPCC definition, other land can also include unmanaged land areas that do not fall into any of the other five land-use categories, for example, heathland, other wooded land (i.e. land with sparse tree cover on mineral soil), and open areas (Norwegian Environment Agency et al. 2020). Coastal and freshwater ecosystems are not assessed.

In addition, the extent and accuracy of ecosystem mapping and carbon budget assessment varies. In the case of forest, an ecosystem type that covers ca 38% of the land surface (Fig. 1), the NFI (Breidenbach et al. 2020) provides systematic accounts of the standing stocks of living trees. The inventories are skewed geographically, with lower density of inventory plots in areas with alpine characteristics, yet these data are the basis of calculating carbon stored in living tree biomass in Norway. For soil and deadwood carbon estimates, the Yasso07 model is used (Liski et al. 2005), parameterized with Norwegian data collected in 1988–92 (de Wit and Kvindesland 1999; Grønlund et al. 2010), and partly with data from elsewhere (de Wit et al. 2006; Dalsgaard et al. 2016). The accounting does not include small trees, shrubs or any other understorey vegetation. Neither does it include the variation in soil biomass, yet Norwegian forest soils hold 3–4 times more carbon than the biomass of the forest trees (Søgaard et al. 2019). Dead wood represents a carbon dense organic matter, most of which currently occurs in forest reserves, which cover only 5% of the forest area in Norway (Norwegian Environment Agency et al. 2019). Despite the forest carbon budget being strongly affected by forestry practices, their impacts on emissions and uptake are not properly accounted for, since a significant part of the relevant carbon stocks and the processes affecting them are not incorporated in the accounting.

Most wetland areas, excluding those used for peat extraction and flooded lands caused by human constructed dams, are considered unmanaged, and therefore not included in the UNFCCC reports (Grassi et al. 2018). For all land categories—forests, cropland, grassland, wetlands, settlement, other land—carbon accounts are reported based on estimated emissions and removals following change in land use between categories. The changes are based on area representative statistics collected as part of the NFI methodology (Norwegian Environment Agency et al. 2020).

The LULUCF category “other land” covers approximately 45% of the total land area in Norway. Much of this is alpine and cryosphere ecosystems that contribute with approximately 13% and 12%, respectively, to the carbon stocks in Norway (Table 1; Fig. 1). However, they are roughly considered as one homogeneous ecosystem, using pooled and coarsely estimated data of carbon stocks and fluxes (Norwegian Environment Agency et al. 2020). The large variety of ecosystems and habitat types within the ‘alpine’ zones results in varied primary production, carbon sequestration and storage levels, that reflect a highly heterogeneous area of land, and one that is particularly vulnerable to climate change, management practices, and land-use conversion (Strimbeck et al. 2019; Bartlett et al. 2020). These accounting conditions may critically misguide climate mitigation measures proposed for the LULUCF sector.

Despite soil holding the highest carbon stocks in boreal forest (Scharlemann et al. 2014; Søgaard et al. 2019), the high costs of measuring soil carbon stocks, uptake and emissions have made it challenging to include forest soil degradation on emissions accounts, thereby also excluding forest soil protection and restoration as an eligible ecosystem component for climate mitigation actions (von Unger and Emmer 2018). Further, the requirement of additionality of climate mitigation actions (i.e. quantitative attribution of carbon removals to specific climate actions under the Clean Development Mechanism, UNFCCC 1998), has led to high reliance and trust on data such as changes in the standing stocks of living trees. While the standing stock of living trees is a convenient statistic, it is not a representative measure for a whole ecosystem.

Of the carbon stocks reported by the official Norwegian accounting system of the LULUCF sector, 90% are based on rough estimates with unknown precision (Table 1).

## Cross-sectoral integration of climate measures on biodiversity—the case of forest fertilization

Forest fertilization is one of the key measures proposed in *Climate Cure 2030*, meaning that knowledge of nitrogen fertilization’s influence on ecosystem GHG fluxes is now crucial for climate action. However, the long-term consequences of fertilizer use on soil communities, that in turn influence nutrient cycling, soil productive capacity, and GHG emissions (Li et al. 2019), are insufficiently known, especially in interaction with the effects of climate change (Coucheney et al. 2013). Simultaneously, excess nitrogen itself is a major global environmental challenge, causing significant biodiversity and ecosystem-service loss both in terrestrial and aquatic systems (Rockström et al. 2009). Although there have been important efforts to reduce the application of mineral fertilizers in many countries (e.g. in the EU), targets to reduce the amount of nitrogen fertilizer in nature have not been achieved (i.e. poor achievement of Aichi target 8.2, "Excess nutrients not detrimental (to nature)", IPBES 2019). In Norway, disputed evidence on the effects of nitrogen fertilization in forests have been summarized to inform *Climate Cure 2030* measures (Box 5; Fig. 3, Aarrestad et al. 2013; Haugland et al. 2014).

With the goal of enhancing atmospheric carbon removals (to compensate for emissions) by15–17 Mt CO_2_-e by the year 2020, forest fertilization was singled out as a promising climate mitigation action for the LULUCF sector in Norway. It was estimated that annual forest fertilization of 120 km^2^ would increase carbon removals by 0.4 Mt over a period of 10 years (Norwegian Ministry of Agriculture and Food 2009). The first national climate change mitigation program, *Climate Cure 2020* (Norwegian Climate and Pollution Agency 2010), recommended fertilization for increasing tree biomass based on these calculations. Initially, there was potential for an integrative governance process: The political backing of using forest fertilization as a climate change mitigation measure was reinforced by a 2012 white paper supported by the Norwegian parliament, while acknowledging that environmental criteria had to be developed (Norwegian Ministry of the Environment 2012, p. 159).

### Marginalization of the value of nature and biodiversity

**Fig. 2:**
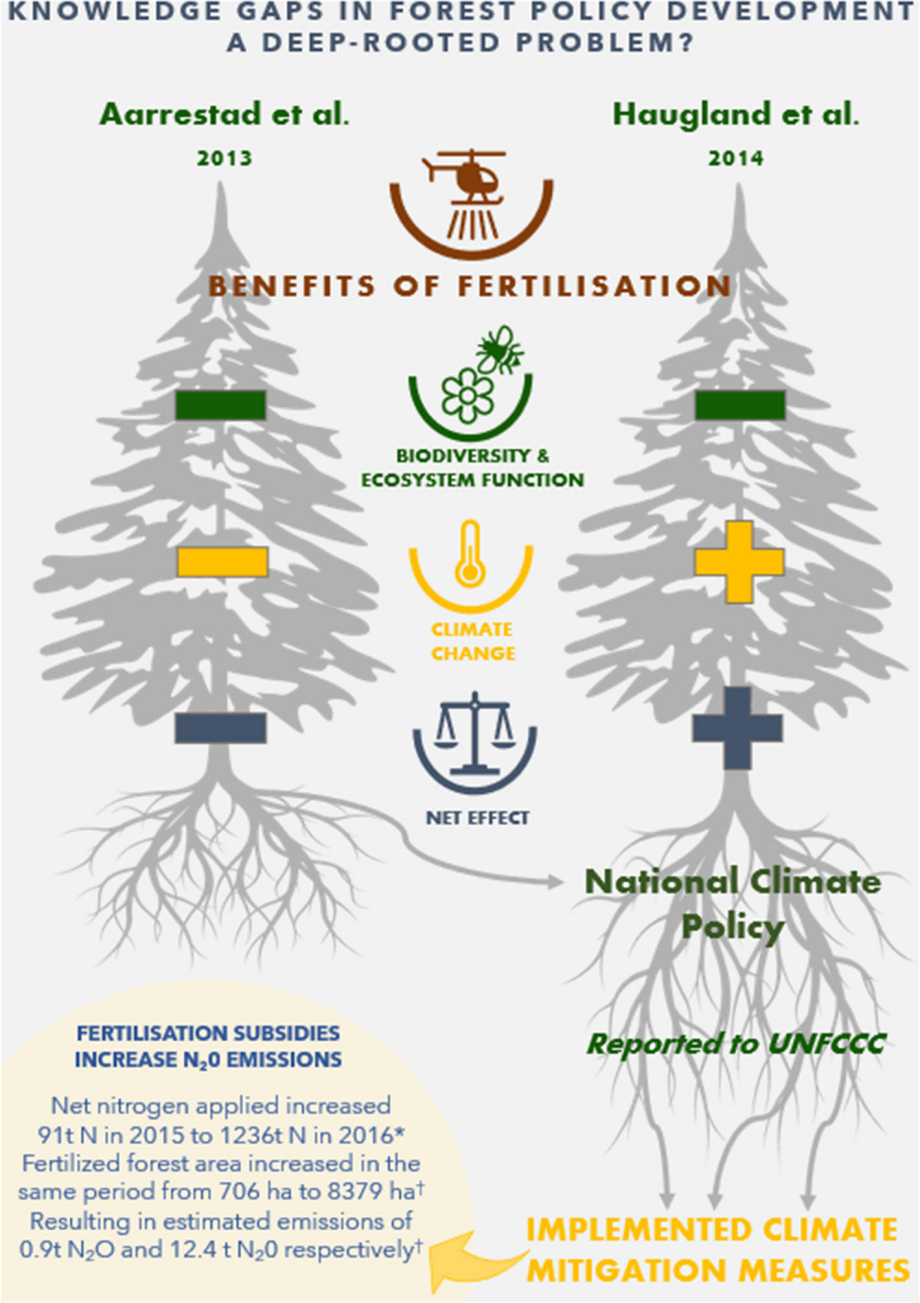
Infographic summarizing the disparate conclusions of two commissioned reports into the effects of forest fertilization (Aarrestad et al. 2013 vs Haugland et al. 2014), and the subsequent influence of the latter report on climate policy, a tenfold increase in forest fertilization application and subsequent impact on greenhouse gas emissions (N_2_O). *Norwegian Environment Agency et al. 2020, and †Statistics Norway 2016

Two reports reviewing the evidence on the effects of nitrogen fertilization on biodiversity (Aarrestad et al. 2013; Haugland et al. 2014) were commissioned to help implement an environmentally friendly climate policy, suggesting an integrative and pluralist approach. Aarrestad et al. (2013) warned against the use of forest fertilization because of the questionable climate effect, considering all GHGs and the high likelihood of very negative effects on biodiversity above and below ground. In contrast, a report published by a broad group of environmental and forestry agencies (Haugland et al. 2014) concluded that the effects on biodiversity would be acceptable, despite citing Aarrestad et al. (2013). The conclusion was based on several assumptions, the main postulate being that fertilization would be used in areas where logging was already planned, and that the effects of fertilization on the individual forest stand would be small compared to the effects of logging (Haugland et al. 2014). The potential effects of fertilization on soil carbon dynamics, as well as the potential for increased N_2_O emissions, were recognized in the report, but left out of the calculations because of uncertainty regarding the exact effects. Hence, regardless of the concerns raised in Aarrestad et al. (2013), Haugland et al. (2014) and later reports (Hanssen and Bergsaker 2017) concluded that the effects of fertilization on terrestrial ecosystems were acceptable, and the net climate effect was evaluated to be positive (Fig. 2).

In the political processes on matters concerning forest fertilization, Haugland et al. (2014) were granted authority over Aarrestad et al. (2013) (Fig. 2). This is perhaps not surprising, as the former had the mandate to conduct a cross-sectoral and integrated review of knowledge and policy options. However, its conclusions established the marginalization of biodiversity and soil carbon as the norm in the following governance processes related to forest fertilization. The report by Haugland et al. (2014) was also the basis for the establishment of climate mitigation subsidies for forest fertilization in 2016, and the only report referenced in the national budget that granted the funding (Norwegian Ministry of Food and Agriculture 2015). The subsidies led to more than a tenfold increase in fertilization; the net amount of nitrogen applied to forest land went from 91  t in 2015 to 1236 t in 2016 (Norwegian Environment Agency et al. 2020, p. 415), and the fertilized forest area increased in the same period from 706 to 8379 ha (Statistics Norway 2020) with estimated emissions of 0.9 t N_2_O and 12.4 t N_2_O, respectively (Norwegian Environment Agency et al. 2020, p. 415).These subsidies were also reported as evidence of climate mitigation action and policy to the UNFCCC (Norwegian Ministry of Climate and Environment 2020) (Fig. 2). Additionally, the report by Haugland et al. (2014) was used as the authoritative source for an evaluation of the climate effects of forest fertilization in 2015 (Andersen et al. 2015), and for climate mitigation actions in the second period of *Climate Cure 2030* (Norwegian Environment Agency 2020). Forest fertilization came out as a leading climate action in these inventory/political reports.

## One climate-and-nature cure

The processes and single-goal approach in addressing the problem of GHG emissions described above have precluded a transformative governance development, as described in Visseren-Hamakers et al. (2021). Specifically, governance approaches have not been *pluralistic* (due to the use of limited data sources and a narrow range of evidence to inform large scale land-use decisions), have shown weakly *integrative* goals across policy sectors (i.e. climate change mitigation and biodiversity conservation), and have not been *inclusive* (with conversational bias limiting which voices are heard). This is despite the initial intention of a cross-sectoral environmental solution. In fact, the goal of *Climate Cure 2030* was to develop a climate action plan with low environmental impact, rather than a land-use plan with joint climate change mitigation, climate adaptation and biodiversity conservation solutions.

Focusing on only one criterion at a time hampers our understanding of the importance of biodiversity and ecosystem function to generate multiple benefits, including the stabilization of the global climate, water flow regulation, erosion control, and protection against natural hazards such as flood, drought and pest outbreaks. It is also unlikely to provide the most effective, or even a positive, solution to climate change in the long term. Pluralist and integrative approaches, addressing multiple considerations including biodiversity, climate change mitigation and costs, would give high outcomes for all co-benefits when optimized (Strassburg et al. 2020). These objectives could be achieved if the most updated evidence about the capacity of Norwegian ecosystems to remove atmospheric GHG could inform joint climate and nature management actions, including a better understanding of the impacts of land-use and management practices on GHG removals and emissions.

**Fig. 3:**
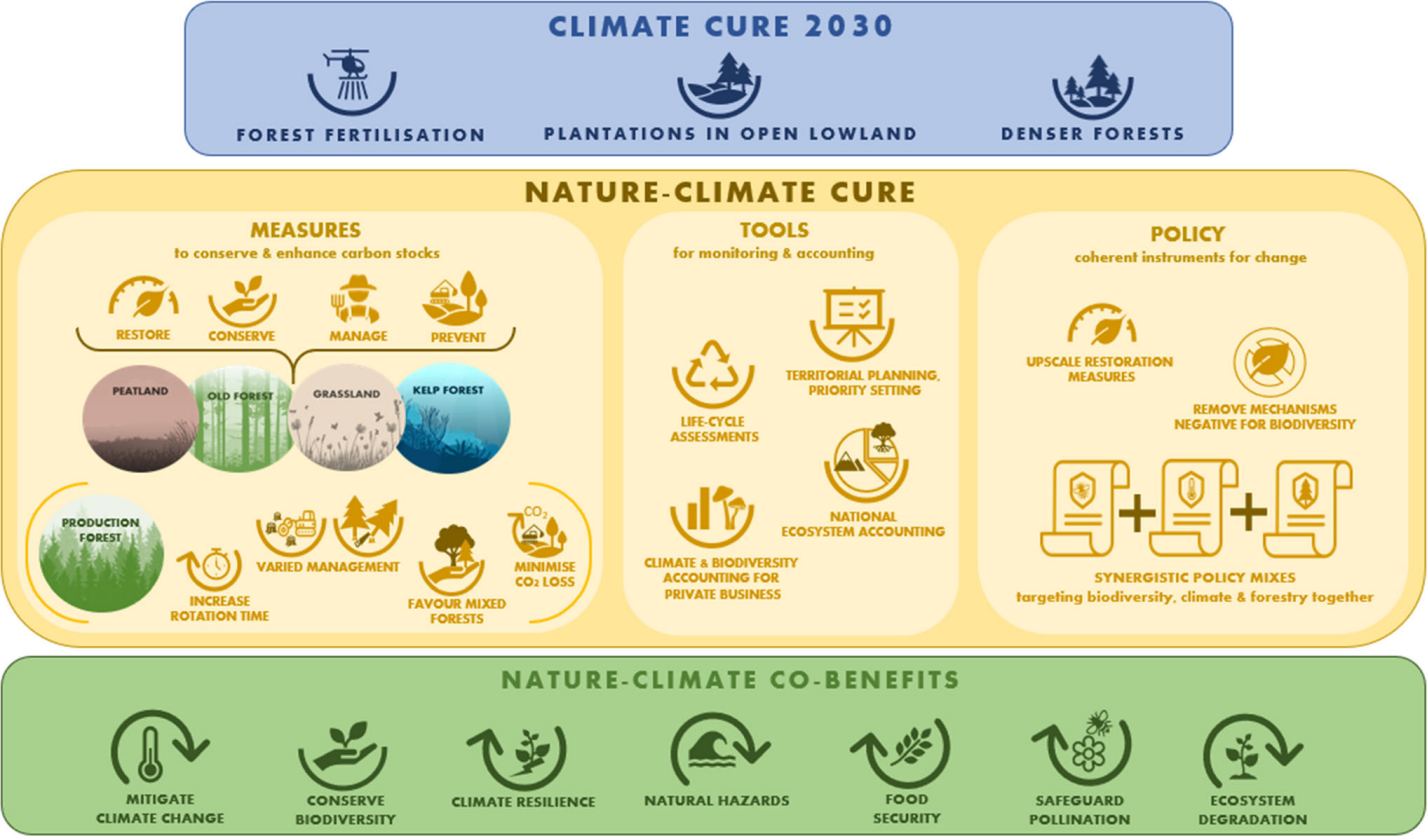
The key points of the *Climate Cure 2030*, and suggestions by the current authors on a holistic alternative in the ‘Nature-climate Cure’, with suggestions for measures, tools and policy instruments that have the potential to mitigate both climate change, and biodiversity loss simultaneously. ‘Measures’ focuses on: key ecosystems that hold both high levels of biodiversity and carbon stocks, and the key measures needed to protect and/or restore them; and necessary measures for inclusion in forestry that will benefit both climate and biodiversity, namely increased rotation times (Stokland et al. 2021), a variety of management practices (with a high share of continuous-cover forestry; Eyvindson et al. 2021), favoring mixed species forests, and avoiding physical disturbance of the soil to minimize GHG loss. ‘Tools’ refers to means to implement joint biodiversity conservation and climate mitigation actions including: spatial targeting of measures to optimize co-benefits (territorial planning); accounting of the effects on biodiversity conservation and climate mitigation of sectoral production processes (life-cycle assessments), and by mainstreaming biodiversity and carbon accounts in economic decision-making, in national statistics (System of Environmental Economic Accounts), and for non-economic disclosures in business reports (Climate and Biodiversity Accounting for Businesses). We highlight the co-benefits of such a joint effort

Nature-climate actions could be developed along three axes that could improve governance approaches towards sustainability: improving ecosystem condition (i.e. of degraded ecosystems by restoration), enhancing transparency and accountability, and fostering sectoral integration and harmonization of goals. These would include measures to protect and enhance carbon stocks, tools for monitoring and accounting, and policy instruments to trigger the adoption of climate and biodiversity solutions (Fig. 3). We provide examples for each of them below.

### I - Natural climate solutions: measures to protect and enhance natural carbon stocks and biodiversity based on a pluralist governance approach

*Climate Cure 2030* is meant to deliver GHG removals within a decade (2021–2030). In this context, and in terms of achieving the Paris Climate Agreement’s goals, the conservation and enhancement of natural GHG sinks and stocks are currently the most efficient and cost-effective actions to take (Villa and Bernal 2018). Such nature-based measures can provide a considerable amount (up to 37%; Griscom et al. 2017) of CO_2_ mitigation needed to meet the targets of the Paris Climate Agreement, but require a systemic understanding of the role of the biosphere in GHG cycles based on an inter-disciplinary pluralist knowledge base. These natural climate solutions also require an integrative governance approach across sectors, since they encompass the fields of land conservation, ecosystem restoration and improved land management actions, which are beneficial for other ecosystem services, such as conserving biodiversity, food security, and reducing the risk of natural hazards (Griscom et al. 2017; IPBES 2019). However, the proposed LULUCF measures in *Climate Cure 2030* neglect these options to a large extent, because they are based on data, models and paradigms of carbon sources and sinks as perceived by the forestry sector alone. Box 2 gives examples of measures that would benefit both climate mitigation and biodiversity conservation (see also Fig. 3).

Box 2. Natural climate solutions^1^. ***Measures to protect and enhance natural carbon stocks and biodiversity.***

- **1. Protect soil organic carbon**. Soil organic carbon (SOC) is a “common denominator” for climate change mitigation initiatives at global and national levels^2^ but is generally overlooked for such measures in Norway^3^. The rapid loss of SOC through the degradation of carbon-rich ecosystems takes decades or even millennia to restore^4,5^. The large land cover of carbon-rich ecosystems such as mires, grasslands and heathlands in Norway presents a huge opportunity to conserve carbon stocks and avoid emissions in Norway^6^ (Fig. 3). In analogy to terrestrial soils, marine sediments are extremely carbon-rich. Protection from bottom trawling would reduce marine CO_2_ emission^7^.
- **2. Reduce forestry harvest rates, increase forestry rotation times, maintain continuous-cover forestry (CCF), and protect forest**. Reduced harvest levels and longer rotation times^8^ have been called for to substantially cut carbon emissions quickly, as requested by IPCC ^9^ and increase ecosystem carbon stocks^10^ These practices together with forest conservation measures, increase the opportunities for spreading and establishment of species that need old forest as a habitat^11^.
- **3. Ecological restoration is part of Norway’s international commitments (CBD, Aichi Biodiversity Target 15)**. Carbon and diversity rich Norwegian marine ecosystems, such as kelp forests and seagrass meadows could be increased by 4.5 Mt C in 30 years^12^. Degraded ecosystems can also be a large source of carbon emissions with drained peatlands in Norway contributing to ca. 10% of Norway’s emissions in 2013, for example^13^. Likewise, considerable climate and biodiversity objectives could be achieved by restoring carbon-rich natural forest ecosystems, i.e. large and old trees, dead wood (especially large logs), mixed tree stands, varied forest structure (age and size of trees), varied structure and species composition in the understorey vegetation, and vegetation cover on the forest floor^14,15^.
- **4. Avoid measures that reduce the area and quality of open habitats**. The lowering of surface albedo accelerates climate-warming feedbacks and should be considered in any climate change mitigation strategy^16^ with low albedo plantations and encroachment areas less beneficial than open, treeless meadows and heaths that have a higher albedo. This will also benefit light-demanding biodiversity. Compared with tree planting, the restoration and proficient management of open ecosystems such as hay-meadows and grasslands will have large positive effects on biodiversity conservation. It would also contribute to achieve specific biodiversity conservation objectives in Norway, such as those formulated in the National Pollinator Strategy and in the plan for the removal of alien species with high ecological risk^17,18^.

**1.** Griscom, B.W., et al. 2017. Natural climate solutions. *PNAS*: 11645–11650. **2.** Bossio, D.A., et al. 2020. The role of soil carbon in natural climate solutions. *Nat. Sustain* 3: 391–398. **3.** Norwegian Ministry of Climate and Environment (2020). Status report as of January 2020; Norway’s fourth Biennial Report under the UNFCCC. **4.** Lange, M., et al. 2015. Plant diversity increases soil microbial activity and soil carbon storage. *Nature Communications* 6: 6707. **5.** Page, S.E., Baird, A.J. 2016. Peatlands and Global Change: Response and Resilience. *Ann. Rev. Env. Res* 41: 35–57. **6.** Bartlett, J., et al. 2020. Carbon storage in Norwegian ecosystems. NINA Report 1774. Norwegian Institute for Nature Research. **7.** Sala et al. 2021. Protecting the global ocean for biodiversity, food and climate. *Nature* 592 (7854): 397–402. **8.** Stokland 2021. Volume increment and carbon dynamics in boreal forest when extending the rotation length towards biologically old stands. *Forest Ecol. Manage.* 488: 119017 **9**. IPCC. 2019. Summary for Policymakers. In: Climate Change and Land: an IPCC special report on climate change, desertification, land degradation, sustainable land management, food security, and greenhouse gas fluxes in terrestrial ecosystems [Shukla, P.R., et al. (eds.)] World Meteorological Organization, Geneva, Switzerland. **10.** Lundmark, T., et al. 2013. Carbon balance in production forestry in relation to rotation length. *Canad. J. Forest Res.* 48: 672–678. **11.** Nordén, J., et al. 2018. At which spatial and temporal scales can fungi indicate habitat connectivity? *Ecol. Indicators* 91: 138–148. **12.** Gundersen, H., et al. 2011. CO_2_ uptake in marine habitats – an investigation (in Norwegian). Norwegian Institute of Water Research, Report 6070, Oslo, Norway. **13.** Joosten, H., et al. 2015. Methods to estimate changes in greenhouse gas emissions following rewetting of peatlands (in Norwegian, English summary). *Naturhistorisk rapport* (10): 1–83. **14.** Janowiak, M., et. al. 2017. Considering forest and grassland carbon in land management. United States Department of Agriculture, Forest Service, General Technical Report WO-95, Washington, D.C., USA. **15.** Wardle, D., et al. 2012. Linking vegetation change, carbon sequestration and biodiversity: insights from island ecosystems in a long-term natural experiment. J. Ecology 100:16–30. **16.** Perugini, L., et al. 2017. Biophysical effects on temperature and precipitation due to land cover change. *Environ. Res. Lett*. 12: 053002. **17.** The Norwegian Ministries 2018. National Pollinator Strategy. A strategy for viable populations of wild bees and other pollinating insects. Oslo, Norway. **18.** Norwegian Ministry of Climate and Environment. 2019. Bekjempelse av fremmede skadelige organismer. Tiltaksplan 2020–2025. 66 pp. Oslo, Norway.

**II - Improving integrative and adaptive governance approaches with tools for planning, monitoring and accounting**

***Avoid carbon emissions from land degradation through spatial planning*** Territorial planning and management, for instance at municipality or county level, is in essence an integrative cross-sectoral governance approach. Provided that maps of ecosystem qualities and their capacity to generate ecosystem services are available, actions can be targeted to optimize multiple functions, and to avoid severe damage. The location of climate mitigation measures (e.g. tree planting, restoration of carbon stocks) is decisive for how effective the measures will be, especially because different localities have different conditions for carbon emissions and storage, which are in turn affected by the local socio-economic context. Brown (2020) recently examined areas with tree planting and mire restoration in the UK and found that the areas where climate measures had been implemented did not necessarily provide the highest net carbon storage capacity. Therefore, spatial planning is important for implementing measures in an effective way. Spatial targeting of actions can also help to achieve multiple objectives and render co-benefits (e.g. to achieve higher carbon uptake, reduce emissions and avoid degradation of nature). There are several tools that have been used for this type of spatial planning in Norway to identify locations where the benefits are optimized, and costs minimized (Schröter et al. 2014; Hanssen et al. 2018). Further, avoiding unnecessary anthropogenic impacts can, among other things, protect against soil erosion and the spread of diseases (see references in Bartlett et al. 2020).

***Life-cycle assessments*** are useful tools for monitoring and reporting effects of measures that have been proposed under the LULUCF category, thus improving transparency, flexibility and adaptive capacity to reorient measures. Examples are "VegLCA" developed for the Norwegian Road Administration (Hammervold 2017) and the Scottish Government’s Peatland Carbon Calculator (Nayak et al. 2010), both including carbon loss from ecosystem degradation caused by development of infrastructure. The latter is used to calculate whether development of renewable energy in a peatland area will give net carbon emission savings, and therefore aims to inform decisions on where to locate wind farms to optimize net carbon removals. In Norway, many carbon-rich ecosystems such as mires and heaths are under pressure from land-use change, e.g. infrastructure and recreation facilities development (e.g. mountain cabins), and it is urgent to apply life-cycle assessment tools or carbon calculators in future planning of such projects to avoid and minimize emissions. The Norwegian Environment Agency has developed a simple spreadsheet to estimate carbon emissions from land-use change, aiming at municipalities working on spatial planning. However, updated knowledge from Norwegian ecosystems, e.g. simple measurements on peat depths, are needed to improve the accuracy of these tools, and there is still no consistent method for including the impacts on biodiversity (Lillesand et al. 2017 and references therein).

***Ecosystem accounting*** has been pointed out as a key tool to benchmark and monitor the status of ecosystems as a response to drivers of change, and to actions that mitigate their negative impacts on biodiversity (IPBES 2019; Turnhout et al. 2021). During the preparation of this manuscript, the System of Environmental Economic Accounting—Ecosystem Accounting (SEEA EA) of the United Nations Statistical Commission laid out a set of principles for measuring the extent of ecosystems, their condition and their capacity to generate ecosystem services that can be used to track the impacts of economic activities on ecosystems and ecosystem services. Specifically, the longer-term aim of implementing these new metrics is to mainstream “the use of the SEEA in policy, including climate change, circular economy, sustainable finance, and biodiversity policy” and “to engage in the monitoring framework of the post-2020 global biodiversity agenda” (United Nations Statistical Commission 2021).

The implementation of SEEA EA is currently being tested at EU scale in a collaboration between EUROSTAT, national statistics bureaus and European research organizations. Although the methods for economic accounting are not yet fully developed, the SEEA EA framework offers opportunities to a broader set of applications to inform specific spheres of economic decision-making, for instance by enabling reporting according to corporate social and environmental responsibility standards (European Commission 2020). In the future, it could be linked to, for instance, EU’s Taxonomy for sustainable activities (Platform for Sustainable Finance 2021).

**III - Improved integrative governance through synergistic instruments**

Upscaling restoration measures that allow for sustainable land management to protect carbon stocks, ecosystem functions and services needs better instruments. There are currently few tools to support ecological restoration in Norway (Olsen et al. 2020). Multiple reinforcing and synergistic mixes of policy instruments (Barton et al. 2017) across sectors would enhance biodiversity, carbon removal functions and other ecosystem services. They could include environmental schemes such as direct payments to landowners, comparable to agri-environmental schemes. These could be implemented relatively quickly, e.g. to start and maintain carbon restoration measures on private land, and to compensate for costs associated with biodiversity and carbon-stock conservation measures (Olsen et al. 2020), including forest cover conservation practices and/or the increase in the length of the logging rotations (Lundmark et al. 2018, references in Bartlett et al. 2020). At a later stage, direct payments could be integrated with new complementary instruments, such as habitat off-setting schemes and standards to evaluate restoration actions, that could be linked to the ecosystem-condition accounts and reporting in the SEEA EA framework (Maes et al. 2021), but that are not currently implemented in Norway. The combination of instrument mix development and analysis with spatial planning tools further provides opportunities to improve the effectiveness of instruments through spatial targeting (Barton et al. 2013, and section II). Additionally, these schemes could be strengthened by combining measures that promote, for instance, the development of new products from restored ecosystems, such as timber from restored deciduous forests (Nordén et al. 2019), with measures to increase the competence of landowners and/or professionals about nature restoration techniques. These measures have a potential to foster a transition towards new economic opportunities for the LULUCF sector, including the forestry sector.

## Conclusions

The uncertainties and data quality biases described above have to a large degree influenced the relative importance attached to the different carbon stocks in nature, as well as which measures are eligible for managing and enhancing atmospheric carbon removals. For instance, soils are the primary carbon store in all Norwegian ecosystems, and their stocks and emissions are highly impacted by land use practices from all sectors; still, soils have been consistently excluded from climate change mitigation actions, despite the huge potential of soil conservation and restoration as an atmospheric GHG removal measure (Morriën et al. 2017; von Unger and Emmer 2018). Instead, remarkably, forest fertilization has been promoted in Norway as a climate mitigation action. This despite its potential negative impacts on biodiversity and the environment, and the fact that Norway is signatory of the Gothenburg Protocol, which aims at reducing the release of nitrogen in nature (UNECE 1999). Our analysis brings to light how transformative changes have the potential to improve the formulation and implementation of synergistic options for biodiversity conservation and climate change actions if considering: (i) a broader and less skewed knowledge base about the biodiversity impacts of land-based climate-mitigation measures, (ii) a more pluralist approach to the value of Norwegian carbon stocks and their changes, and (iii) an improved cross-sectoral dialogue and integration. These can be fostered by concerted actions including a re-direction of economic mechanisms (McElwee et al. 2020), enabling regulations and new metrics to quantify both positive and negative impacts on climate and biodiversity (Turnhout et al. 2021).

## Supplementary Information

Below is the link to the electronic supplementary material.

Supplementary file1 (PDF 253 kb)
