# Supplementary material for: A joint climate and nature cure: A transformative change perspective
Source: Ambio. 2022 Jan 25;51(6):1459–73. doi: 10.1007/s13280-021-01679-8 (PMC9005584; doi:10.1007/s13280-021-01679-8)
Supplement: Supplementary file 1 — Supplementary file1 (PDF 253 kb) [file 13280_2021_1679_MOESM1_ESM.pdf]

Ambio

Supplementary Information

This supplementary information has not been peer reviewed.

Title: **A joint climate and nature cure: A transformative change perspective**

Authors: Graciela M. Rusch, Jesamine Bartlett, Magni Olsen Kyrkjeeide,
Ulrika Lein, Jenni Nordén, Hanno Sandvik, Håkon Stokland

Supplement: Data underlying Table 1

This table summarises the data underlying Table 1 and Figure 1.

Ecosystem / area type	Subtype	Carbon density (kg C m^{-2})	Carbon density reference	Area (km^2)	Area reference	Carbon storage (Mt C)
Built-up/urban		n/a		5500	SSB (2019)	n/a
Bare rock		n/a		26 000	Bryn et al. (2018)	n/a
Glaciers/ice		0.009	Anesio et al. (2009)	2700	NVE (2019)	0.025
Permafrost		58	Hugelius et al. (2014)	13 000	Gisnås et al. (2016)	750
Alpine				104 800		800
	nival zone	1.0–4.6	estimates from Tibet (Ohtsuka et al. 2008); assuming sedge and grass snow beds in nival zones, after Bryn et al. (2018), are more similar to alpine meadows after Sørensen et al. (2017); cf. Post et al. (1982)	19 500	Bryn et al. (2018)	20–90
	Shrubs	6.6	Sørensen et al. (2017)	39 000	Bryn et al. (2018)	260
	Heaths	9.2	Sørensen et al. (2017)	38 000	Bryn et al. (2018)	350
	Meadows	12	Sørensen et al. (2017)	8300	Bryn et al. (2018)	100
Forests				121 000	SSB (2019)	2300
	living tree biomass (aboveground)	4	Søgaard et al. (2019)			500
	understorey biomass	0.2	based on estimates for Sweden and Finland (Nilsson and Wardle 2005; Muukkonen and Mäkipää 2006; Wardle et al. 2012)			23

Ecosystem / area type	Subtype	Carbon density (kg C m^{-2})	Carbon density reference	Area (km^2)	Area reference	Carbon storage (Mt C)
	soil, including dead wood and litter	15	Søgaard et al. (2019); Pregitzer and Euskirchen (2004) and Tomter and Dalen (2018) suggest lower estimates			1800
Cultivated land				11 700		100
	grassland	9.8	Norwegian Environment Agency (2019)	2300	SSB (2019)	22
	cropland	8.3	Norwegian Environment Agency (2019)	9400	SSB (2019)	78
Heathlands				6800		103
	coastal	9.3	estimates for sandy soils from UK (Milne and Brown 1997; Alonso et al. 2012)	2700	Bryn et al. (2018)	25
	other	17–21	estimates for podsols from UK (Milne and Brown 1997)	4100	Bryn et al. (2018)	78
Wetlands				24 000		1400
	undisturbed mires	50–280	Cannell et al. (1993); Grønlund et al. (2010); higher estimates from UK, Evans et al. (2016)	17 000	Joosten et al. (2015); Bryn et al. (2018)	870–2100
	disturbed mires	5	assuming 10% of undisturbed	3600–7000	Joosten et al. (2015)	20
Freshwater				18 000	NVE (2018)	900
	biomass	0.003	Cyr and Peters (1996)			0.05
	sediments	50	extrapolation based on sedimentation rate in Sweden (Algesten et al. 2003), which is compatible with flux data from Norway (Larsen et al. 2011) and with sediment estimates from North America (Alin and Johnson 2007; Munroe and Brencher 2019)			900
Coastal				9370		8.1
	kelp forest	0.45	Gundersen et al. (2011)	8000	Gundersen et al. (2011)	3.6
	seagrass meadow	0.08	estimates for Skagerrak (Röhr et al. 2018)	93	Gundersen et al. (2018)	0.47
	intertidal algae	0.225	Gundersen et al. (2011)	180	Gundersen et al. (2011)	0.04

Ecosystem / area type	Subtype	Carbon density (kg C m^{-2})	Carbon density reference	Area (km^2)	Area reference	Carbon storage (Mt C)
	saltmarsh	20 000	estimates from UK (Chmura et al. 2003)	100	informed guess	2
	intertidal mudflat	2000	estimates from France and UK (Spilmont et al. 2006; Alonso et al. 2012)	1000	informed guess	2

References

Algesten, G., S. Sobek, A.-K. Bergström, A. Ågren, L.J. Tranvik, and M. Jansson. 2003. Role of lakes for organic carbon cycling in the boreal zone. *Global Change Biology* 10: 141–147.

Alin, S.R., and T.C. Johnson. 2007. Carbon cycling in large lakes of the world: a synthesis of production, burial, and lake-atmosphere exchange estimates. *Global Biogeochemical Cycles* 21: GB3002.

Alonso, I., K. Weston, R. Gregg, and M. Morecroft. 2012. Carbon storage by habitat: review of the evidence of the impacts of management decisions and condition of carbon stores and sources. Natural England, Research Report 43, York, U.K.

Anesio, A.M., A.J. Hodson, A. Fritz, R. Psenner, and B. Sattler. 2009. High microbial activity on glaciers: importance to the global carbon cycle. *Global Change Biology* 15: 955–960.

Bryn, A., G.-H. Strand, M. Angeloff, and Y. Rekdal. 2018. Land cover in Norway based on an area frame survey of vegetation types. *Norsk geografisk tidsskrift* 72: 131–145.

Cannell, M.G.R, R.C. Dewar, and D.G. Pyatt. 1993. Conifer plantations on drained peatlands in Britain: a net gain or loss of carbon? *Forestry (London)* 66: 353–369.

Chmura, G.L., S.C. Anisfeld, D.R. Cahoon, and J.C. Lynch. 2003. Global carbon sequestration in tidal, saline wetland soils. *Global Biogeochemical Cycles* 17: 1111.

Cyr, H., and R.H. Peters. 1996. Biomass-size spectra and the prediction of fish biomass in lakes. *Canadian Journal of Fishery and Aquatic Science* 53: 994–1006.

Evans, C., R. Morrison, A. Burden, A., J. Williamson, A. Baird, E. Brown, N. Callaghan, P. Chapman, et al. 2016. Final report on project SP1210: Lowland peatland systems in England and Wales – evaluating greenhouse gas fluxes and carbon balances. S.l.: Centre for Ecology and Hydrology.

Gisnås K., B. Etzelmuller, C. Lussana, J. Hjort, A.K. Sannel, K. Isaksen, S. Westermann, P. Kuhry, et al. 2016. Permafrost map for Norway, Sweden and Finland. *Permafrost and Periglacial Processes* 28: 359–378.

Grønlund A., K. Bjørkelo, G. Hylen, and S.M. Tomter. 2010. CO_2 sequestration of soil and vegetation in Norway. Storage, sequestration and emissions of CO_2 and other greenhouse gases. Bioforsk, Report 5(162), Ås, Norway (in Norwegian).

Gundersen, H., H.C. Christie, H. de Wit, K.M. Norderhaug, T. Bekkby, and M.G. Walday. 2011. CO_2 uptake in marine habitats – an investigation. Norwegian Institute of Water Research, Report 6070, Oslo, Norway (in Norwegian).

Gundersen, H., T. Bekkby, K.M. Norderhaug, E. Oug, and S. Fredriksen. 2018. Seagrass meadow. In *Norwegian Red List for ecosystems 2018*, ed. Norwegian Biodiversity Information Centre, https://artsdatabanken.no/RLN2018/18. Trondheim: NBIC (in Norwegian).

Hugelius, G., J. Strauss, S. Zubrzycki, J.W. Harden, E.A.G. Schuur, C.-L. Ping, L. Schirrmeister, G. Grosse, et al. 2014. Estimated stocks of circumpolar permafrost carbon with quantified uncertainty ranges and identified data gaps. *Biogeosciences* 11: 6573–6593.

Joosten, H., A. Barthelmes, J. Couwenberg, K. Hassel, A. Moen, C. Tegetmeyer, and A. Lyngstad. 2015. Methods to estimate changes in greenhouse gas emissions following rewetting of peatlands. *Naturhistorisk rapport* (10): 1–83 (in Norwegian, English summary).

Larsen, S., T. Andersen, and D.O. Hessen. 2011. Climate change predicted to cause severe increase of organic carbon in lakes. *Global Change Biology* 17: 1186–1192.

Milne, R., and T.A. Brown. 1997. Carbon in the vegetation and soils of Great Britain. *Journal of Environmental Management* 49: 413–433.

Munroe, J., and Q. Brencher. 2019. Holocene carbon burial in lakes of the Uinta Mountains, Utah, USA. *Quaternary* 2: 13.

Muukkonen, P., and R. Mäkipää. 2006. Empirical biomass models of understorey vegetation in boreal for-ests according to stand and site attributes. *Boreal Environment Research* 11: 355–369.

Nilsson, M.-C., and D.A. Wardle. 2005. Understory vegetation as a forest ecosystem driver: evidence from the northern Swedish boreal forest. *Frontiers in Ecology and the Environment* 3: 421–428.

Norwegian Environment Agency. 2019. Greenhouse gas emissions 1990–2017, national inventory report. Norwegian Environment Agency, Report M-1271, Oslo, Norway.

NVE [Norwegian Water Resources and Energy Directorate]. 2018. Lake database. Retrieved 21 September, 2018, from https://www.nve.no/karttjenester/kartdata/vassdragsdata/innsjodatabase/ (in Norwegian)

NVE [Norwegian Water Resources and Energy Directorate]. 2019. Glaciers. Retrieved 22 January, 2020, from https://www.nve.no/hydrology/glaciers/

Ohtsuka, T., M. Hirota, X. Zhang, A. Shimono, Y. Senga, M. Du, S. Yonemura, S. Kawashima, et al. 2008. Soil organic carbon pools in alpine to nival zones along an altitudinal gradient (4400–5300 m) on the Tibetan Plateau. *Polar Science* 2: 277–285.

Post, W.M., W.R. Emanuel, P.J. Zinke, and A.G. Stangenberger. 1982. Soil carbon pools and wild life zones. *Nature (London)* 298: 156–159.

Pregitzer, K.S., and E.S. Euskirchen. 2004. Carbon cycling and storage in world forests: biome patterns related to forest age. *Global Change Biology* 10: 2052–2077.

Röhr, M.E., M. Holmer, J.K. Baum, M. Björk, K. Boyer, D. Chin, L. Chalifour, S. Cimon, et al. 2018. Blue carbon storage capacity of temperate eelgrass (*Zostera marina*) meadows. *Global Biogeochemical Cycles* 32: 1457–1475.

Søgaard, G., M. Allen, R. Astrup, H. Belbo, E. Bergseng, H.H. Blom, R. Bright, L. Dalsgaard, et al. 2019. Effects of afforestation on climate, environment and economy. Norwegian Institute of Bioeconomy Research, Report 5(3), Ås, Norway (in Norwegian).

Sørensen, M.V., R. Strimbeck, K.O. Nystuen, R.E. Kapas, B.J. Enquist, and B.J. Graae. 2017. Draining the pool? Carbon storage and fluxes in three alpine plant communities. *Ecosystems (New York)* 21: 316–330.

Spilmont, N., D. Davoult, and A. Migné. 2006. Benthic primary production during emersion: in situ measurements and potential primary production in the Seine Estuary (English Channel, France). *Marine Pollution Bulletin* 53: 49–55.

SSB [Statistics Norway]. 2019. Land use and land cover. Retrieved 10 February, 2020, from https://www.ssb.no/en/natur-og-miljo/statistikker/arealstat/aar/2019-05-27#content

Tomter, S.M., and L.S. Dalen, eds. 2018. Sustainable forestry in Norway. Ås: Norwegian Institute of Bioeconomy Research (in Norwegian).

Wardle, D.A., M. Jonsson, S. Bansal, R.D. Bardgett, M.J. Gundale, and D.B. Metcalfe. 2012. Linking vegetation change, carbon sequestration and biodiversity: insights from island ecosystems in a long-term natural experiment. *Journal of Ecology* 100: 16–30.
